# How social skills relate to academic life integration: psychological well-being and loneliness

**DOI:** 10.3389/fpsyg.2026.1891012

**Published:** 2026-10-26

**Authors:** Hongyu An, Bojan Obrenovic, Gang Du, Aisulu Dzhanegizova, Danijela Godinić, Xiao Gu

**Affiliations:** 1 School of Communication, Langfang Normal University, Langfang, China; 2 Zagreb School of Economics and Management, Zagreb, Croatia; 3 School of Business and Management, Q University, Almaty, Kazakhstan; 4 Media Literacy Research Institute, Communication University of Zhejiang, Hangzhou, China; 5 Al-Farabi Kazakh National University, Almaty, Kazakhstan; 6 Faculty of Humanities and Social Sciences, University of Zagreb, Zagreb, Croatia; 7 Almaty Humanitarian-Economic University, Almaty, Kazakhstan; 8 Regional Rural Governance Institute, Hangzhou Polytechnic, Hangzhou, China

**Keywords:** academic life integration, cross-sectional study, loneliness, psychological well-being, self-determination theory, social cognitive theory, social skills, university students

## Abstract

**Background:**

This study examined whether social skills relate to academic life integration directly and indirectly through psychological well-being and loneliness.

**Methods:**

A cross-sectional survey of 251 Hangzhou Polytechnic students was analyzed using PLS-SEM.

**Results:**

Social skills were positively associated with psychological well-being and ALAS and negatively associated with loneliness. The indirect association through psychological well-being was significant (*β* = 0.176, 95% CI 0.099−0.271); the indirect association through loneliness was not (*β* = 0.028, 95% CI −0.006−0.060; *p* = 0.090). The primary model explained 43.0% of ALAS variance.

**Conclusion:**

The psychological well-being pathway remained significant across examined specifications; support for the loneliness pathway was sensitive to specification. Weak convergent validity, limited model fit, and sampling limitations warrant cautious interpretation.

## 1 Introduction

Contemporary higher education is marked by substantial transformation in learning formats and opportunities for interpersonal interaction (Sá, 2023). Institutions need to leverage diverse formats to provide a student-centered, customized learning experience. Institutional success depends on students’ learning outcomes and their ability to leverage social, communication, critical-thinking, meta-cognitive, goal-setting, and technical skills (Bergmark and Westman, 2018; Brouwer and Jansen, 2019; Sá and Serpa, 2018). Social Skills are a set of behaviors that enable one to communicate effectively with peers across various social contexts. Social experiences are central to institutional integration, as they are critical for meeting the demands of the social and academic contexts (Kahu et al., 2020). The social aspect of academic engagement encompasses interaction between learners, lecturers, institutions, and study content.

Against this background, the literature presents different accounts of how Social Skills relate to Psychological Well-Being (PWB) and academic outcomes. Social competence has a bidirectional relationship with Psychological Well-Being (Gomez-Lopez et al., 2022), and Social Skills are linked to well-being and academic performance (Arhuis-Inca and Ipanaqué-Zapata, 2023). Other studies examine intervening processes, such as perceived stress (Segrin et al., 2007) and positive interpersonal relationships (Segrin and Taylor, 2007). More specifically, Bhargava and Sharma (2024) examined Psychological Well-Being as a mediator in the relationship between teamwork skills and student engagement. Therefore, these individual relationships are not new. Prior research has examined loneliness as a mediator between Social Skills and mental-health outcomes among university students (Moeller and Seehuus, 2019). Loneliness has also been linked to learning engagement in online study environments (Mizani et al., 2022; Ramli et al., 2024). The unresolved question addressed here is whether Psychological Well-Being and loneliness each account for a distinct indirect association between Social Skills and academic life integration when considered jointly. Testing them in parallel prevents treating positive psychological functioning and low loneliness as interchangeable explanations.

Social Cognitive Theory (SCT) and Self-Determination Theory (SDT) provide complementary perspectives on these relationships. SCT provides an account of interpersonal competence, behavior, and access to social resources (Bandura, 1986), whereas SDT explains the relevance of Relatedness, Competence, and Autonomy to Positive Functioning (Deci and Ryan, 2000). Together, they link interpersonal behavior with positive psychological functioning and perceived social disconnection, supporting the two indirect pathways rather than treating low loneliness as equivalent to well-being.

This study aims to empirically examine whether Social Skills are associated with loneliness and Psychological Well-Being. Moreover, we analyze their relationships with academic life integration, measured by the Academic Life Assessment Scale (ALAS), and examine the two specific indirect associations. ALAS assesses perceived academic ability, involvement in non-compulsory activities, and commitment to the course (dos Santos et al., 2020). It therefore concerns the broader appraisal of university life rather than engagement alone. Whereas much of the existing literature has focused on academic engagement and motivation or on institutional factors (Zajda, 2024; Liang et al., 2025), we examine whether positive functioning and perceived social disconnection account for distinct associations with this broader outcome in a predominantly first-year sample at one Chinese institution. The contribution lies in this joint, context-specific examination, with sensitivity analyses assessing whether the conclusions depend on the specification of the PWB measure.

This question is relevant to counselors, student affairs professionals, instructional designers, and health promotion specialists seeking to support students’ Psychological Well-Being and motivation to learn.

## 2 Literature review and theoretical framework

### 2.1 Social skills and their role in academic and emotional functioning

Social proficiency encompasses communication and collaboration, empathy and perspective-taking, self-regulation, teamwork, active listening and assertiveness, all of which support healthy group dynamics, cooperative learning, participation, negotiation and conflict resolution. Students scoring highly in social competency are more likely to integrate seamlessly into peer groups, affiliations, and mentorship programs, and to exhibit strong communication and collaborative potential. Moreover, skillful interpretation of social cues and emotional regulation improve conflict resolution and enable individuals to deal with misunderstandings effectively. Therefore, it is likely they will benefit from available institutional and social resources that improve coping capacity. Advanced socio-emotional abilities can significantly improve peer relationships, foster empathy and perspective-taking, and strengthen social bonds (Syed and Sri, 2023). Furthermore, teacher, peer, family, and institutional support, along with constructive feedback, increase engagement (Bowles et al., 2022).

Social Skills draw on attentional, communication, and socio-emotional regulation abilities, all of which are crucial for successful and advantageous peer interaction, group participation, and classroom engagement. Socio-emotional competency can strengthen relationships by fostering empathy, perspective-taking, emotional intelligence, and emotional regulation, reducing reactivity and the risk of detachment or withdrawal during disagreements. These abilities may support adherence to academic norms, perseverance, commitment, and participation in formal and informal academic activities. Moreover, they have been found to increase engagement (Kiema-Junes et al., 2020) and affect academic outcomes, such as performance and achievement (Haneef, 2024).

Behaviors such as communication, regulation, and collaboration underlie adherence to social norms and, in turn, promote Psychological Well-Being. Interpersonal skills are honed through solution-focused interventions that emphasize improving communication, cooperation, empathy, and self-control. Additionally, such programs substantially enhance relationships between students and educators (Niu et al., 2022).

### 2.2 Loneliness and academic life integration

Loneliness concerns a perceived discrepancy between desired and actual social relationships, rather than merely a lack of contacts (Russell et al., 1978). It is taxing on a subjective level, as it involves feelings of alienation, isolation, and exclusion. Reduced participation can limit access to peer relationships and support, with implications for Psychological Well-Being (Dinu et al., 2022; Kaufmann and Vallade, 2022). In line with SDT (Deci and Ryan, 1985), loneliness may reflect unmet relatedness needs. This provides a rationale for expecting a negative association with students’ involvement in university life, without assuming that the association is the same across settings.

Recent research has examined loneliness and academic engagement, particularly during the transition to higher education and shifts in peer networks (Bevens et al., 2024; Cole et al., 2021). Loneliness is commonly assessed using the University of California, Los Angeles (UCLA) Loneliness Scale (Russell et al., 1978). Published studies link Perceived Loneliness to students’ well-being (Dinu et al., 2022; Kaufmann and Vallade, 2022) and to their educational experience (Stoliker and Lafreniere, 2015).

Despite the theoretical relevance of the relationship between loneliness and student engagement, its association with broader academic-life outcomes warrants examination. We therefore assess the association between loneliness and ALAS conditional on Social Skills and Psychological Well-Being, rather than assuming that lower loneliness necessarily accompanies greater academic integration.

Engagement, as presupposed in active learning, implies students’ participation in problem-solving, discussions, peer activities, simulations and practical applications. Loneliness has been empirically shown to adversely affect Psychological Well-Being (Dinu et al., 2022), engagement (Ramli et al., 2024), learning experience (Kaufmann and Vallade, 2022), and performance (Singh et al., 2021; Mizani et al., 2022). Social relationships buffer against loneliness, particularly among young adults, and support adaptive coping through Self-Acceptance and realistic expectations of oneself and significant others (Schoenmakers and ten Bruggencate, 2024).

### 2.3 Psychological well-being and the indirect pathway

Psychological Well-Being is a multifaceted construct with six core dimensions: Self-Acceptance, Positive Relationships, Autonomy, Personal Growth, Purpose in Life, and Environmental Mastery (Ryff, 1989). That is, Psychological Well-Being entails positive self-appraisal, growth and development, goal setting and achievement, stable interpersonal relationships, the ability to self-regulate and manage one’s life and surroundings, and independence and self-determination. Psychological Well-Being may be associated with academic involvement through Environmental Mastery, Resilience, and Purposefulness, although these processes should not be equated with intrinsic motivation. It is associated with life satisfaction, fulfillment of social and relatedness needs, Self-Acceptance, and emotional balance. Social ties, including peer relationships and teacher or mentor support, reinforce a sense of belonging, enhancing both social and academic efficacy, and, in turn, strengthening identification and belonging.

Well-being is linked to psychological fulfillment, a sense of coherence, meaningful engagement, and vitality. Moreover, it is based on higher self-efficacy, a Sense of Autonomy, agency, and control (Zheng et al., 2020). It includes positive emotions and a favorable appraisal of one’s quality of life when basic social needs are met (Bidzan-Bluma et al., 2020). Adaptive functioning and Resilience arise when students can relate to one another, proactively engage, and enjoy teamwork (Lorber et al., 2023). Psychological well-being, therefore, refers to Resilience and balance across mental, emotional, affective, and social well-being. One essential component is interpersonal well-being, reflecting positive subjective experiences of social connectedness and emotional closeness that arise in academic and social contexts, peer affiliations, teams, friendships, and mentorships (Hatun, 2025).

According to SDT, satisfying the needs of Autonomy, Competence, and Relatedness supports well-being and autonomous motivation (Deci and Ryan, 2000). Perceived Control and Autonomy reinforce a positive Self-Concept, Improving Self-Actualization, Clarity, Focus, Persistence, and Resilience. However, this relationship can be hindered by perceived rejection, exclusion, or lack of acceptance, which can lead to loneliness and reduce the desire to participate in relevant academic group activities. In contrast, good Social Skills and social efficacy improve relationship management and conflict resolution, enabling students to take control across social contexts, maintain Resilience under pressure, and retain focus and adaptability in stressful situations. Therefore, as anticipated by SCT, emotional regulation helps reduce anxiety and distress, supports emotional balance, and buffers against the effects of isolation and alienation on academic engagement, while also aiding coping and conflict resolution. Although relationships between Social Skills and engagement have been studied, Psychological Well-Being and loneliness may represent distinct pathways linking Social Skills to the integration of academic life.

### 2.4 Student engagement and academic life integration

Student engagement refers to a positive outlook toward academic, social, extracurricular, and learning activities, as well as interest, meaning, goal attainment, and enjoyment (Bergdahl et al., 2020; Zhao et al., 2021). More specifically, academic involvement will be partially determined by a favorable assessment of the institutional and social climate and by active student voice, whereby individuals perceive that they are involved in decision-making and that institutions value their input, efforts, and opinions. Social engagement will rely on advantageous peer interactions, willingness to engage in collaborative learning, formal and informal participation, and adequate social integration, with the central feature a sense of belonging. The emotional dimension of student engagement focuses on academic interest, enjoyment of learning, growth mindset, positive involvement, and achievement motivation, all of which strengthen commitment. In addition, students’ appraisals of study material and teaching methodology affect their engagement. Namely, when lecturers are appraised as unapproachable, detached, unrelatable, uninterested, or unable to provide feedback and support (Lodge et al., 2018), or when the content appears overly abstract, unrelated, confusing, and unmanageable, learning motivation will decrease. Learning motivation includes agentic engagement, task engagement, and self-regulated learning, in which individuals are willing to exert mental effort and use strategic approaches to understand and connect complex ideas. Contextual, institutional, and motivational factors influence engagement. Prior research has identified teaching practices and institutional support as key predictors of engagement.

In an asynchronous online course in Chinese higher education, Hu et al. (2023) examined self-regulated learning as a mediator linking social and cognitive presence with learning satisfaction. In a broader sample of Chinese universities, Zhang et al. (2023) found that self-regulated learning mediated the association of teaching and cognitive presence with online learning satisfaction, but not the association between social presence and satisfaction. In a different academic setting, Li et al. (2023) linked stress to doctoral performance through self-regulated learning. These studies examine distinct psychological pathways and outcomes rather than directly testing the present social-skills model. Previous research has consistently shown a positive, persistent relationship between academic and social integration (Browning et al., 2018; Xerri et al., 2018).

According to SDT, engagement is related to autonomous motivation, in which an individual’s internal values and interests prompt all behavior. When the needs for Autonomy, Competence, and Relatedness are met, students are more willing to engage in learning processes. Equally important, Social Skills underscore relatedness and competence. Lower Perceived Loneliness may therefore be linked to stronger social integration.

Previous studies have examined students’ learning engagement and its impacts on academic outcomes (Lei et al., 2018; Yoon et al., 2020; Bergdahl et al., 2020). As mentioned before, active participation in formal and informal extracurricular activities increases both agentic engagement and investment in learning, involvement, persistence, motivation, and commitment (Niu et al., 2025; Driessen et al., 2020).

ALAS assesses students’ perceived integration into academic life (dos Santos et al., 2020), including academic ability, involvement in non-compulsory activities, and commitment to the course. We therefore use academic life integration for the measured outcome.

### 2.5 Research model development

SCT explains why communication, cooperation, and access to social resources may connect Social Skills with Loneliness and Psychological Well-Being (Hypotheses H1 and H2), as well as with participation in university life (Hypothesis H6). SDT complements this account by relating Autonomy, Competence, and Relatedness to positive functioning and academic involvement (Hypotheses H3 and H4) (Bandura, 1986; Deci and Ryan, 2000). Together, these perspectives motivate the parallel indirect associations through Psychological Well-Being and Loneliness (Hypotheses H5a and H5b). These theories have also informed research on supervisor support and graduate-student creativity (Li et al., 2025). Ryff’s PWB dimensions operationalize positive functioning, not SDT need satisfaction. Self-efficacy, resource use, and need satisfaction inform the rationale but are not separately measured mediators in this model.

#### 2.5.1 Relationship between social skills and loneliness

Social Skills pertain to social competence, the ability to understand and relate to others, engage in cooperative behaviors, and manage relationships, misunderstandings, and social differences. They facilitate communication, bonding, and adaptability, key features of relatedness, and dimensions of Psychological Well-Being and Resilience. Moreover, they enable individuals to achieve seamless integration and group cohesion, encouraging prosocial behavior, empathy, understanding, and identification. Aside from improving social, interpersonal, relational, and emotional well-being, they stimulate adaptive coping, encourage help-seeking, and support the exchange and utilization of resources, thereby enhancing Resilience, persistence, and balance. Communication and perspective-taking may help resolve conflicts and foster cooperation, strengthening a sense of belonging. These skills are critical for successful social engagement and, when executed, facilitate the establishment of a vast social network and participation in peer group activities. They were previously linked to attachment styles and past experiences (DiTommaso et al., 2003). Loneliness is a negative valence, qualitative, or quantitative assessment of one’s social network (Bugay, 2007). Such negative appraisals are especially relevant and common during the transitional period from adolescence to young adulthood, often accompanied by the transition from school to university life. Leaving the old social network and forming new connections, an intermediary process in which students are briefly isolated from peers, places significant strain and causes substantial acute loneliness (Moeller and Seehuus, 2019; Bruffaerts et al., 2018; Cheng and Furnham, 2002).

Previously, loneliness was associated with attachment styles because insecure individuals have negative self-perception and views of others, and exhibit severe skill imbalance and/or deficit (DiTommaso et al., 2003). Loneliness was associated with low self-esteem and poor social relations (Panayiotou et al., 2016). Such traits perpetuate social rejection and exacerbate biases in assessing social threat. Individuals without adequate social models and reinforcement fail to develop social competence. Demanding and intense academic and social contexts may be stressful and taxing for personal, social, and relational well-being. Thriving students are expected to exhibit Autonomy, Environmental Mastery, Goal-Achievement Motivation, and Emotional Endurance. Social Skills play a significant role in social awareness, management, and adaptability (Moeller and Seehuus, 2019). Failing to understand others, take different perspectives, or empathize may result in a lack of interpersonal sensitivity and lead to conflict, rejection, and alienation. More specifically, students with poor Social Skills will experience interpersonal difficulties and fail to establish stable relationships, thereby failing to make the majority of the of available social and institutional support. Consequently, they are at risk of disengaging, detaching, experiencing emotional instability, and more often experiencing loneliness (Danneel et al., 2018; Richardson et al., 2017).

Furthermore, poor Social Skills and low emotional regulation increase susceptibility to loneliness and the tendency toward depression (Fauziyyah and Ampuni, 2018). Therefore, we present Hypothesis H1:

*H1*: Social skills are negatively associated with loneliness.

#### 2.5.2 Social skills and psychological well-being

Psychological Well-Being, with its related concepts of Self-Acceptance, Personal Growth, Purpose, and Positive Relationships, is directly related to Social Skills. In line with SDT, Social Skills facilitate the fulfillment of basic psychological needs for Autonomy, Competence, and Relatedness by empowering individuals to form meaningful relationships, seek and receive social support, and engage in beneficial, reciprocal, trust-based relationships. The presence of social engagement and strong social bonds is a known protective factor against psychological distress. In the same vein, effective relationship management and social adaptability enhance Feelings of Competence and Autonomy, which, in turn, increase Psychological Well-Being. Empirical evidence primarily supports this direct relationship. A study investigating the impact of Social Skills on Loneliness (Dharun and Neelakandan, 2024) found that engaging in social interactions and the quality of relationships have a substantial negative effect on adverse psychological outcomes and are likely to manifest in the future social adaptability. Moreover, a series of recent studies has indicated that Social Skills can reduce Loneliness, social anxiety, and related adverse psychological consequences among adolescents and young adults (Parenteau et al., 2024). Similarly, multiple systematic studies have shown that participation in The Program for the Education and Enrichment of Relational Skills (PEERS) substantially increases social knowledge, skills, and social awareness (Zheng et al., 2021). Curran and Elwood (2024) integrated the Social Skill deficit vulnerability model with basic psychological needs theory to examine interpersonal communication apprehension and loneliness. In line with SDT, students experience Autonomy as a sense of overall satisfaction and integrity in their actions, thoughts, and feelings (Deci and Ryan, 2000), which are carried out without external pressure. Furthermore, individuals with strong Social Skills are more proficient in social interactions and more confident in their mastery.

Additionally, Social Skill deficiencies lead to poorer relational quality and reduced social adaptation, both of which are directly linked to adverse sociopsychological well-being. Individuals with impaired social competence have difficulties in socialization and interpersonal communication, are apprehensive about their social roles, and are likely to experience fear and anxiety in social situations. Emotional intelligence, a component of interpersonal competencies, is positively related to positive psychological characteristics and improved Psychological Well-Being (Shengyao et al., 2024). Therefore, we present Hypothesis H2:

*H2*: Social Skills are positively associated with Psychological Well-Being.

#### 2.5.3 Loneliness and academic life integration

A vast body of literature has corroborated the association between loneliness and student engagement. Prior studies have mostly linked loneliness to various learning behaviors, including engagement, achievement, sense of belonging, and Psychological Well-Being (Mizani et al., 2022). Ramli et al. (2024) examined the negative association between loneliness and student engagement, as well as the role of academic self-efficacy. These findings provide a basis for examining the corresponding association with ALAS.

Loneliness, as a consequence of reduced social connectedness and relationship instability, can translate into low academic integration due to diminished social integration and social capital. Social embeddedness allows individuals to draw on their ties, affiliations, and networks and to use social, emotional, and institutional resources to buffer against stress and anxiety. Academic and transitional stressors may have severe consequences for well-being (Bruffaerts et al., 2018; Moeller and Seehuus, 2019). Moreover, Social Skills improve coping, adaptability, regulation, and Resilience, as well as the willingness to face challenges and recover from setbacks (Ramli et al., 2024). When emotional balance is disrupted, cognitive clarity, focus, and persistence decrease, and students experience low self-esteem, emotional dysregulation, diminished agency and mastery, and negative self-appraisal. They tend to detach from both social and academic situations they perceive as unmanageable or threatening. Feedback and support from peer groups can significantly increase self-esteem and efficacy. In contrast, lonely individuals may exhibit low tolerance for anxiety and stress, high detachment, a diminished sense of meaning and purpose, and even an unstable sense of identity.

According to SDT, relatedness is important for autonomous motivation (Deci and Ryan, 2000). Perceived disconnection may therefore be associated with reduced participation in university activities. Therefore, we propose Hypothesis H3:

*H3*: Loneliness is negatively associated with academic life integration.

#### 2.5.4 Psychological well-being and academic life integration

Psychological Well-Being, with its social and emotional components, increases positive experiences and improves emotional regulation, Resilience, adaptability, and overall optimism, thereby affecting learning, growth, and goal attainment. It supports learning engagement by reducing stress sensitivity and reactivity, improving social and academic integration, and enhancing Environmental Mastery and agency in challenging academic situations. Moreover, a sense of meaning and purpose strengthens task and learning persistence and improves decision-making, problem-solving, creativity, and execution. In line with SCT, Positive Self-Concept, Self-Efficacy, Autonomy, and Competency lead to greater learning capacity, more adaptive coping in taxing social or intellectual academic situations, and better resource procurement and utilization. Psychologically balanced students are better able to navigate and mitigate challenges during the transition to universities by building strong relationships (Samsudin et al., 2024), engaging in prosocial and collaborative behaviors, engaging in intellectual activities, and strategically using social and institutional support resources. In contrast, prior research has established that low Psychological Well-Being is associated with diminished social, communication, empathy, and critical thinking skills (Khairani et al., 2022).

Individuals scoring high on Psychological Well-Being show greater study engagement, better performance, and less avoidance. Moreover, they are autonomous, efficient, and confident, with stronger motivation and greater personal engagement. They persist in the face of challenges, adapt, invest effort, employ constructive learning strategies, and engage in proactive learning (Kotera et al., 2022; Samsudin et al., 2024). Existing research links well-being to academic engagement (Babosalam et al., 2022; Puiu et al., 2024) and to the related outcome of academic achievement (Klapp et al., 2024); student perspectives also inform accounts of school well-being (Powell et al., 2018). Psychological capital was found to predict Psychological Well-Being and student engagement in higher education (Gebregergis et al., 2023).

Accordingly, in addition to formal education, universities should prioritize promoting quality of life and Psychological Well-Being (Gebregergis et al., 2023). Wang and Zhang (2024) have used SCT to explain how self-efficacy and Resilience, as key aspects of well-being, relate to academic engagement. Therefore, we present Hypothesis H4:

*H4*: Psychological Well-Being is positively associated with academic life integration.

#### 2.5.5 Indirect association through psychological well-being

Because Social Skills enable individuals to engage in healthy and enduring social relationships, they increase the use of personal, social, and institutional resources to improve one’s psychological Resilience and buffer against strain on both physical and mental health. Furthermore, Social Skills may be indirectly associated with academic life integration by amplifying the effects of psychological capital and by improving self-efficacy, assertiveness, and relatedness, thereby enhancing motivation, willingness to participate in educational settings, and ultimately engagement (Gebregergis et al., 2023). Quality social interactions enhance well-being by meeting basic psychological needs and fostering positive emotions, which, in turn, promote academic involvement. In line with SDT, the Social Skill components of assertiveness, negotiation, conflict resolution, empathy, and cooperation facilitate the initiation of social interaction, improve social responsiveness, and increase social activity, thereby satisfying individuals’ need for relatedness and improving overall Psychological Well-Being, including intrinsic learning motivation (Wang et al., 2024). Psychological, emotional, and social capital, along with the efficient use of available functional, cognitive, and institutional resources, improve Resilience, emotional regulation, and stress management, thereby increasing learning engagement (Gebregergis et al., 2023). Related research links students’ well-being with academic engagement (Puiu et al., 2024). Therefore, we present Hypothesis H5a:

*H5a*: Social Skills have a positive indirect association with academic life integration through Psychological Well-Being.

#### 2.5.6 Indirect association through loneliness

Loneliness has been shown to adversely affect students’ learning outcomes (Kaufmann and Vallade, 2022). By supporting social engagement, interpersonal communication, and peer involvement, Social Skills may be associated with lower loneliness and greater participation in collaborative academic activities. Peer support is crucial to individuals’ sense of relatedness and correlates with academic engagement (Ulmanen et al., 2024). Withdrawal may serve as a maladaptive coping strategy to reduce social stressors.

Prior studies have found that poor or inadequate peer relationships negatively affect study engagement (Tikkanen et al., 2024). The lower the loneliness, the higher the academic self-efficacy and engagement (Aslan and Polat, 2024). Therefore, we present Hypotheses H5b and H6:

*H5b*: Social Skills have a positive indirect association with academic life integration through lower Loneliness.

#### 2.5.7 Direct association of social skills with academic life integration

The proposed mediators need not account for the entire relationship. Communication and cooperation may also be directly linked to participation in university life.

*H6*: Social Skills are positively associated with academic life integration, conditional on Psychological Well-Being and Loneliness.

Figure 1 presents the hypothesized relationships.

**Figure 1:**
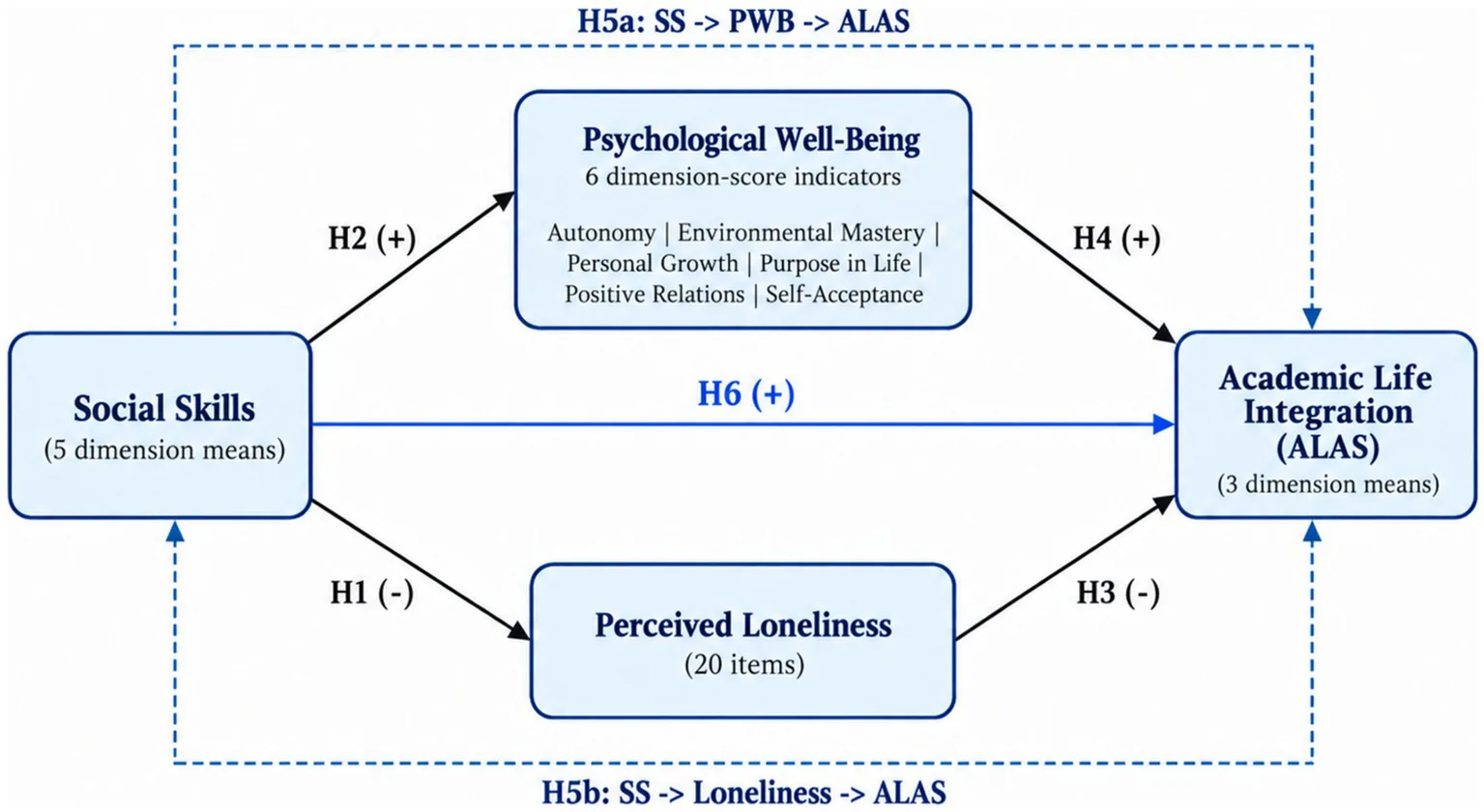
Hypothesized research model.

## 3 Materials and methods

### 3.1 Participants and sampling

Ethical approval was obtained from the appropriate institutional review board. Respondents provided informed consent electronically. The study population consisted of university students enrolled in higher education programs. This group was selected because university students typically experience significant academic, social, and psychological transitions, making them an appropriate population for examining relationships among Social Skills, Loneliness, Psychological Well-Being, and integration into academic life. University settings provide an environment in which students interact socially, adapt to new academic demands, and develop personal competencies, offering a relevant context for investigating the mechanisms underlying academic outcomes and well-being.

Data were collected at Hangzhou Polytechnic in Hangzhou, Zhejiang Province, China, during scheduled class sessions. Students completed the Chinese-language electronic questionnaire in the classroom via WeChat. A total of 275 questionnaires were collected; 24 incomplete questionnaires were excluded during data screening, leaving 251 complete responses for analysis. No missing-value imputation or further case deletion was required for the analysis.

At the structural level, ALAS has three predictors, whereas PWB and loneliness each have a single predictor. The model was estimated using the 251 complete responses, and the precision of the direct and indirect associations was assessed using bootstrap confidence intervals. Sample size alone does not establish adequate power for a small indirect association.

The demographic profile of the sample included largely women (76.9%); 18.7% men; and 4.4% preferred not to say (Table 1). The majority of respondents were aged 18–21 (92.0%), followed by those under 18 (6.8%), and those over 26 (1.2% total). The combined Chinese/Other Category accounted for 99.2% of the sample. When it comes to academic status, the majority were first-year (97.2%), second-year (2.0%), third-year (0.4%), and master’s level (0.4%) students. Students represented Arts and Humanities (57.8%), Engineering (31.1%), Social Sciences (10.8%), and Business Administration (0.4%). The Grade Point Average (GPA) self-reported distribution reveals that the majority of the students performed well academically. About 57.4% reported a GPA of 3.4–4.0. 25.9% reported a GPA of 3.0–3.4. 12.4% reported a GPA of 2.0–2.9. 4.4% reported a GPA below 2.0. Given the sample composition, generalizations beyond this specific student group should be made with caution.

**Table 1:** Demographic profile of the sample.

Demographic characteristics	Category	Frequency	Percentage (%)
Gender	Male	47	18.7
	Female	193	76.9
	Prefer not to say	11	4.4
Age	Under 18 years old	17	6.8
	18–21 years old	231	92.0
	26–29 years old	1	0.4
	30–35 years old	1	0.4
	40 years and above	1	0.4
Ethnicity	Black/African American	2	0.8
	Chinese/other	249	99.2
University year	Year 1 (Freshman)	244	97.2
	Year 2 (Sophomore)	5	2.0
	Year 3 (Junior)	1	0.4
	Master’s student	1	0.4
Study area	Business administration	1	0.4
	Engineering	78	31.1
	Arts and humanities	145	57.8
	Social sciences	27	10.8
GPA	Below 2.0	11	4.4
	2.0–2.9	31	12.4
	3.0–3.4	65	25.9
	3.4–4.0	144	57.4

### 3.2 Measures and instruments

#### 3.2.1 Social skills scale

To evaluate an individual’s ability to navigate social situation scenarios and maintain interpersonal relationships, we used a scale developed by Padhy and Hariharan (2023). The administered adaptation presents 23 statements rated on a 5-point agreement scale, from strongly disagree (1) to strongly agree (5). The scale covers five dimensions: affiliative skills, leadership, team integration, interpersonal skills, and social engagement. Notable items from the scale include “I actively/patiently listen to what people have to say” and “I am good at handling conflicts in a group.”

#### 3.2.2 Perceived loneliness scale

The perceived loneliness scale was developed by Russell et al. (1978) to assess an individual’s subjective experiences of loneliness and social isolation. The administered 20-item adaptation uses five frequency categories, ranging from never (1) to very frequently (5); participants indicated how often each statement applied to them. Higher scores indicate more loneliness. Notable items from the scale include the following: “I am unhappy doing so many things alone,” “I have nobody to talk to,” and “I feel as if nobody really understands me.”

#### 3.2.3 Psychological well-being scale

The psychological well-being scale, derived from Ryff (1989) and Ryff and Keyes (1995), is designed to assess various facets of an individual’s mental well-being. The administered 18-item instrument comprises six theoretical dimensions: Self-Acceptance, Positive Relations, Autonomy, Environmental Mastery, Purpose in Life, and Personal Growth. The administered version uses a 5-point agreement scale from strongly disagree (1) to strongly agree (5). Notable items from this scale comprise: “I like most parts of my personality,” “In general, I feel I am in charge of the situation in which I live,” and “For me, life has been a continuous process of learning, changing, and growth.” Item-retention results and the resulting dimension-score indicators are reported in Section 4.1; dimension-level sensitivity analyses are reported in Section 4.4.

#### 3.2.4 Academic Life Assessment Scale (ALAS)

The Academic Life Assessment Scale (ALAS) was examined by dos Santos et al. (2020) as a measure of students’ integration into the academic environment. Their revised 23-item structure comprises student ability, involvement in non-compulsory activities, and commitment to the course. The administered Chinese questionnaire contains 22 items on a 5-point agreement scale: the published item 10 is absent, and later local item numbers shift by one. All 22 local items contribute to three-dimensional means: ability (9 items), non-compulsory activities (6 items) and course commitment (7 items). Local items 2, 6, 9, 10, 12, 17, 18 and 21 are reverse-keyed as 6 minus the response before averaging. The three-dimensional means were used as reflective indicators of academic life integration.

Across the four instruments, 83 items were administered. Negative items were reverse-keyed as 6 minus the response, and dimensional means were then calculated. Before administration, the questionnaire was translated from English to Chinese and then back-translated into English. Discrepancies were resolved to preserve conceptual equivalence, and the Chinese version was evaluated for face validity, linguistic clarity, and comprehensibility.

### 3.3 Procedure

The Chinese-language online questionnaire began with information about the study, confidentiality, and voluntary participation, followed by demographic questions and multiple self-report instruments. The four instruments used here were part of a larger questionnaire.

The survey assessed Social Skills, Loneliness, Psychological Well-Being, and academic life integration through multi-item measures. Gender, age group, ethnicity, year in university, study area, and self-reported GPA were recorded.

### 3.4 Data analysis approach

The primary SmartPLS 4.1 analysis used a reflective, multidimensional specification with 251 cases and 34 indicators. Indicators were standardized; path weighting used unit initial weights, a convergence criterion of 10^−7^, and a maximum of 3,000 iterations. Inference used 5,000 bootstrap resamples, two-tailed tests at α = 0.05, and 95% percentile confidence intervals (CIs). Coefficients are standardized, and totals use unrounded estimates. Results from the earlier unidimensional item-level analysis are not used to evaluate this specification.

The primary PWB construct retained all six Ryff-dimensional means. Five and four-dimensional models omitted Purpose in Life and then Positive Relations as data-informed sensitivity analyses, using the same cases, structural paths, and estimation settings. Social Skills, PWB, and ALAS were represented by arithmetic dimensional means after reverse-keying and item screening (Section 4.1); loneliness remained a 20-item block. Each bootstrap was conditional on the retained scores and did not repeat item or dimensional selection.

PLS-SEM estimates construct scores from weighted indicators and is appropriate when the analysis emphasizes explained variance in endogenous constructs and the proposed parallel associations (Hair et al., 2022).

### 3.5 Assessment of common method bias (CMB)

Although data were collected from a single source at a single time point using self-report measures, we recognized the potential for common method bias (CMB). Procedural safeguards were consistent with recommendations by Podsakoff et al. (2003). We assured participants that their responses would be kept anonymous and confidential to reduce the likelihood that they would respond positively to appear socially desirable. We separated the predictor variables from the outcome variables in the survey instrument by placing items measuring predictor variables in one section and those measuring the outcome variables in another. The survey instructions emphasized that there were no right or wrong answers, thereby reducing the potential for evaluation apprehension and acquiescence bias among respondents.

Full-collinearity VIFs were 1.944 (SS), 1.224 (PL), 1.875 (PWB), and 1.755 (ALAS), indicating no severe collinearity. These collinearity diagnostics do not rule out common method bias.

## 4 Results

### 4.1 Measurement model

We evaluated the measurement model for reliability and validity following recommended PLS-SEM procedures (Hair et al., 2022). We examined outer loadings (FL), internal consistency (Cronbach’s alpha [CA], rho_A, and composite reliability [CR; rho_C]), convergent validity (average variance extracted; AVE), and discriminant validity (Fornell-Larcker and HTMT) using the primary six-dimensional SmartPLS output and the documented within-dimensional checks (Fornell and Larcker, 1981; Henseler et al., 2015).

Within-dimensional screening excluded PWB05_R, PWB03, PWB04_R, PWB13, PWB14_R, and PWB15_R, which were identified in the refinement record as the weakest items in their respective dimensions. The remaining twelve items were used to create six two-item dimensional means in the primary model. These item-level exclusions preceded the dimension-level sensitivity analyses in Section 4.4.

The resulting primary specification represents 77 of the 83 administered items: all 23 social-skills (SS) items, summarized into 5-dimensional means; all 20 Perceived-Loneliness (PL) items; 12 retained Psychological Well-Being (PWB) items, summarized into 6-dimensional means; and all 22 ALAS items summarized into 3-dimensional means. It therefore contains 34 model indicators. Item screening retained representation of each theoretical PWB domain, but did not independently validate the shortened subscales.

As a rule of thumb, loadings should be above 0.70, although lower loadings may be retained when theoretically important and interpreted alongside CR and AVE (Hair et al., 2022). In the primary six-dimensional model, the dimension-score loadings ranged from 0.766 to 0.836 for ALAS, from 0.001 to 0.847 for Psychological Well-Being, and from 0.828 to 0.940 for Social Skills. The individual-item loadings for Loneliness ranged from 0.642 to 0.838.

Three retained loneliness loadings are below 0.70: LON01 (0.642), LON03 (0.661), and LON06 (0.693). For the six PWB indicators, Autonomy (0.719), Personal Growth (0.754), Environmental Mastery (0.822), and Self-Acceptance (0.847) loaded moderately to strongly. Positive Relations was weak (0.431, *p* < 0.001), while Purpose in Life was effectively unrelated to the higher-order PWB construct (0.001, *p* = 0.992). Thus, the primary six-dimensional specification preserves theoretical breadth but does not provide strong measurement support for all six dimensions. This pattern motivated the five and four-dimensional sensitivity models (Table 2).

**Table 2:** Reliability and convergent validity.

Construct/indicator	FL	CA	rho_A	CR	AVE
Academic life assessment scale		0.705	0.725	0.833	0.625
ALAS_ABILITY	0.836				
ALAS_ACTIVITY	0.766				
ALAS_COMMIT	0.767				
Loneliness		0.966	0.969	0.968	0.607
LON01	0.642				
LON02	0.809				
LON03	0.661				
LON04	0.813				
LON05	0.771				
LON06	0.693				
LON07	0.808				
LON08	0.801				
LON09	0.838				
LON10	0.818				
LON11	0.824				
LON12	0.744				
LON13	0.776				
LON14	0.837				
LON15	0.784				
LON16	0.770				
LON17	0.780				
LON18	0.766				
LON19	0.820				
LON20	0.788				
Psychological well-being (6 dimensions)		0.686	0.796	0.793	0.444
PWB_AUTONOMY	0.719				
PWB_GROWTH	0.754				
PWB_MASTERY	0.822				
PWB_PURPOSE	0.001				
PWB_RELATIONS	0.431				
PWB_SELFACC	0.847				
Social skills		0.934	0.940	0.950	0.792
SS_AFFIL	0.876				
SS_ENGAGE	0.909				
SS_INTER	0.940				
SS_LEAD	0.894				
SS_TEAM	0.828				

For the primary model, ALAS, Loneliness and Social Skills showed internal consistency estimates above 0.70. For PWB, Cronbach’s alpha was 0.686, slightly below 0.70, while rho_A = 0.796 and composite reliability = 0.793 exceeded 0.70. The very high loneliness reliability (alpha = 0.966; CR = 0.968) warrants attention to possible item redundancy. Convergent validity was adequate at the modeled level for ALAS (AVE = 0.625), Loneliness (AVE = 0.607), and Social Skills (AVE = 0.792), but not for the six-dimensional PWB construct (AVE = 0.444). Thus, convergent validity of the primary six-dimensional PWB construct was limited.

In the primary model, each square root of AVE exceeded the corresponding inter-construct correlations (Table 3), and HTMT ranged from 0.356 to 0.760 (Table 4), below 0.85. These discriminant-validity results do not remove the PWB convergent-validity limitation. Figure 2 summarizes the primary structural model.

**Table 3:** Fornell-Larcker discriminant validity criterion.

	ALAS	PL	PWB	SS
ALAS	0.791			
PL	−0.334	0.779		
PWB	0.565	−0.398	0.666	
SS	0.606	−0.349	0.621	0.890

**Table 4:** Heterotrait-monotrait ratio of correlations (HTMT).

	ALAS	PL	PWB	SS
ALAS				
PL	0.391			
PWB	0.760	0.455		
SS	0.720	0.356	0.699	

**Figure 2:**
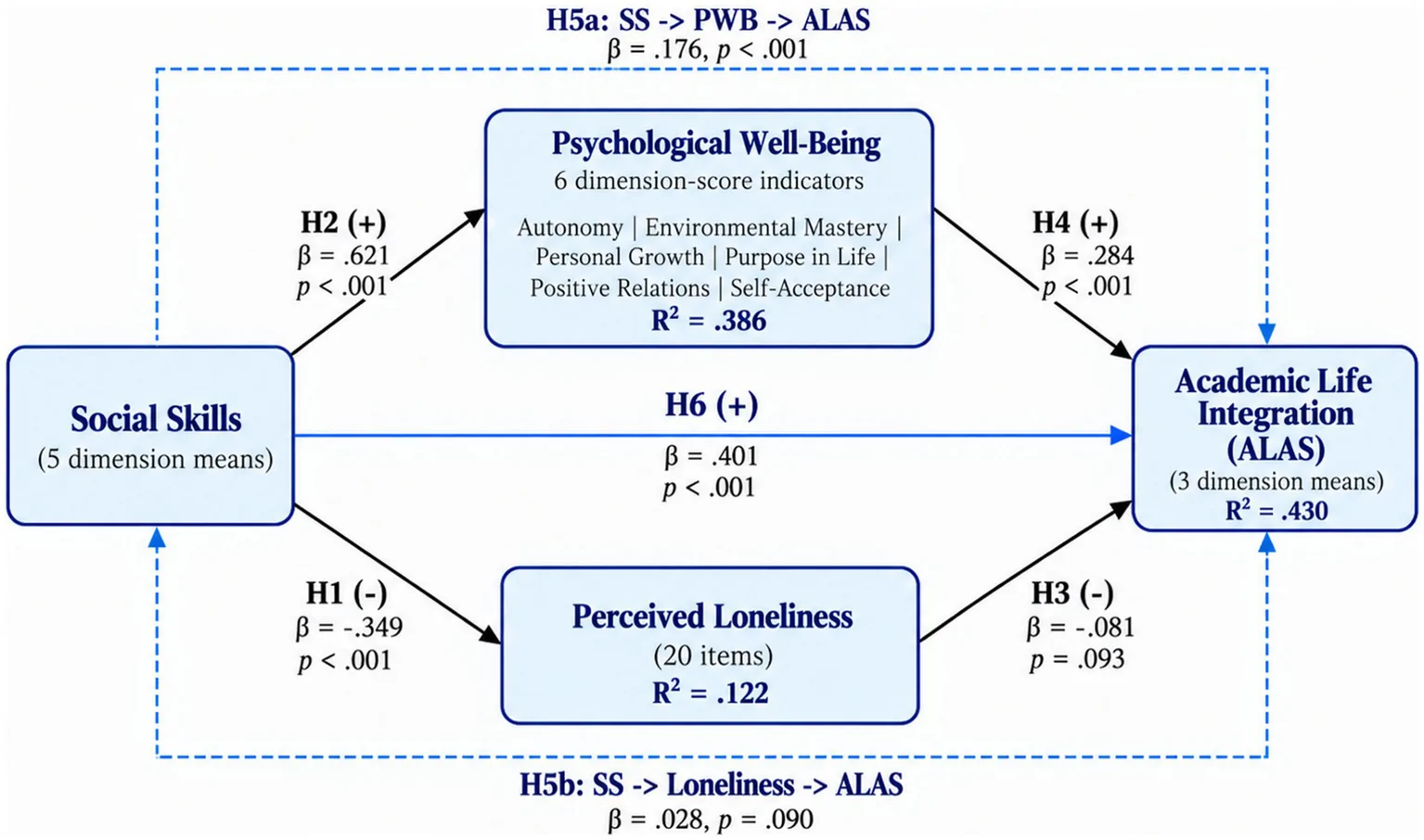
Primary six-dimensional PWB structural model.

### 4.2 Structural model assessment

The primary predictors of ALAS showed no severe collinearity: VIFs ranged from 1.212 to 1.734, with a minimum tolerance of 0.577 (Table 5; Hair et al., 2022).

**Table 5:** Collinearity of the primary six-dimensional PLS predictors of ALAS.

Predictor	VIF	Tolerance	Collinearity problem
PWB	1.734	0.577	No
SS	1.662	0.602	No
PL	1.212	0.825	No

The structural model’s explanatory power (*R*^2^), effect sizes (*f*^2^), and collinearity were analyzed in accordance with accepted PLS-SEM reporting guidelines (Hair et al., 2022). *R*^2^ values represent in-sample explained variance.

The primary structural model explained 43.0% of the variance in academic life integration (ALAS: *R*^2^ = 0.430; adjusted *R*^2^ = 0.423), 38.6% of the variance in Psychological Well-Being (PWB: *R*^2^ = 0.386; adjusted *R*^2^ = 0.384), and 12.2% of the variance in Perceived Loneliness (PL: *R*^2^ = 0.122; adjusted *R*^2^ = 0.118). Social Skills, therefore, account for only part of the variation in loneliness; this *R*^2^ value does not, by itself, explain the nonsignificant loneliness-to-ALAS path.

We assessed *f*^2^ to determine the contribution of each predictor to an endogenous construct (Cohen, 1988; Hair et al., 2022). In the primary model, effects on ALAS were negligible for PL > ALAS (*f*^2^ = 0.010), small for PWB > ALAS (*f*^2^ = 0.081), and moderate for SS > ALAS (*f*^2^ = 0.170). SS showed a large effect on PWB (*f*^2^ = 0.629) and a small effect on PL (*f*^2^ = 0.138).

Tables 6, 7 report the standardized coefficients, two-tailed *p*-values, and 95% percentile bootstrap intervals for the direct and indirect associations.

**Table 6:** Direct associations in the primary six-dimensional PWB model.

Relationship	Coefficient (*β*)	95% CI	Bootstrap SE	*t*-ratio	*p*-value
H3: PL- > ALAS	−0.081	[−0.173, 0.015]	0.049	1.678	0.093
H4: PWB- > ALAS	0.284	[0.164, 0.413]	0.063	4.486	<0.001
H6: SS- > ALAS	0.401	[0.293, 0.499]	0.053	7.578	<0.001
H1: SS- > PL	−0.349	[−0.465, −0.238]	0.058	6.048	<0.001
H2: SS- > PWB	0.621	[0.533, 0.711]	0.045	13.815	<0.001

**Table 7:** Specific indirect, total indirect, and total associations.

Label	Relationship	Estimate (*β*)	95% CI	*p*-value	Decision
Total indirect	SS- > ALAS via both mediators	0.205	[0.127, 0.298]	<0.001	Interval excludes 0
H5a	SS- > PWB- > ALAS	0.176	[0.099, 0.271]	<0.001	Supported
H5b	SS- > PL- > ALAS	0.028	[−0.006, 0.060]	0.090	Not supported
Total	Direct + total indirect	0.606	[0.533, 0.676]	<0.001	Interval excludes 0

In the primary model, Social Skills were negatively associated with loneliness and positively associated with PWB and ALAS. PWB was positively associated with ALAS, whereas the conditional loneliness-to-ALAS association was nonsignificant. Thus, Hypotheses H1, H2, H4, and H6 were supported, but H3 was not (Table 6).

The indirect association through PWB was significant [*β* = 0.176, 95% CI: (0.099, 0.271), *p* < 0.001], supporting H5a; the loneliness pathway was not [*β* = 0.028, 95% CI: (−0.006, 0.060), *p* = 0.090], leaving H5b unsupported. The total indirect and total associations were also significant (Table 7).

### 4.3 Model fit

The primary six-dimensional model had Standardized Root Mean Square Residual (SRMR) = 0.085 (saturated) and 0.092 (estimated). The bootstrap-based exact-fit assessment was unfavorable: SRMR, d_ULS, and d_G exceeded their 95th and 99th-percentile reference values for both models. These results indicate limited global fit.

### 4.4 Sensitivity analyses of five and four-dimensional PWB reductions

#### 4.4.1 Specifications and measurement properties

The five-dimensional model omitted Purpose in Life; the four-dimensional model also omitted Positive Relations, retaining Autonomy, Personal Growth, Environmental Mastery, and Self-Acceptance. PWB was represented by 10 and eight underlying items, respectively. These models contained 33 and 32 indicators, representing 75 and 73 questionnaire items, and used the same 251 cases and structural paths as the primary model (Table 8).

**Table 8:** PWB measurement comparison across the primary and sensitivity specifications.

Measure	Six dimensions (primary)	Five dimensions (sensitivity)	Four dimensions (sensitivity)
Model indicators	34	33	32
Underlying PWB items	12	10	8
Autonomy loading	0.719	0.720	0.761
Personal growth loading	0.754	0.752	0.770
Environmental mastery loading	0.822	0.824	0.843
Purpose in life loading	0.001	Omitted	Omitted
Positive relations loading	0.431	0.426	Omitted
Self-acceptance loading	0.847	0.850	0.844
PWB Cronbach’s alpha	0.686	0.763*	0.820
PWB rho_A	0.796	0.797*	0.831
PWB composite reliability	0.793	0.845*	0.880
PWB AVE	0.444	0.533*	0.648
PWB *R*^2^	0.386	0.387*	0.359

The five-dimensional Positive Relations loading remained weak (0.426). In the four-dimensional model, PWB loadings ranged from 0.761 to 0.844, Composite Reliability (CR) = 0.880, and Average Variance Extracted (AVE) = 0.648 (Table 8). HTMT ranged from 0.347 to 0.720, with all 95% upper bounds below 0.85. These improvements concern the narrower, retained dimension-score construct, not validation of all 18 original PWB items.

The five-dimensional reliability and R^2^ values marked with an asterisk in Table 8 are taken from the model-comparison record; these statistics were not included in the model-comparison record’s bootstrap export.

#### 4.4.2 Structural associations and explained variance

Figure 3 illustrates the four-dimensional model; Table 9 compares all structural associations and their 95% percentile intervals. The PWB indirect association remained significant across the six-, five-, and four-dimensional models (0.176, 0.174, and 0.145). The loneliness indirect association was nonsignificant in the five-dimensional model but significant in the four-dimensional model [0.037, 95% CI: (0.005, 0.069), *p* = 0.021], as was loneliness → ALAS [−0.107, 95% CI: (−0.196, −0.014), *p* = 0.022]. The total SS → ALAS association remained approximately 0.606–0.607.

**Figure 3:**
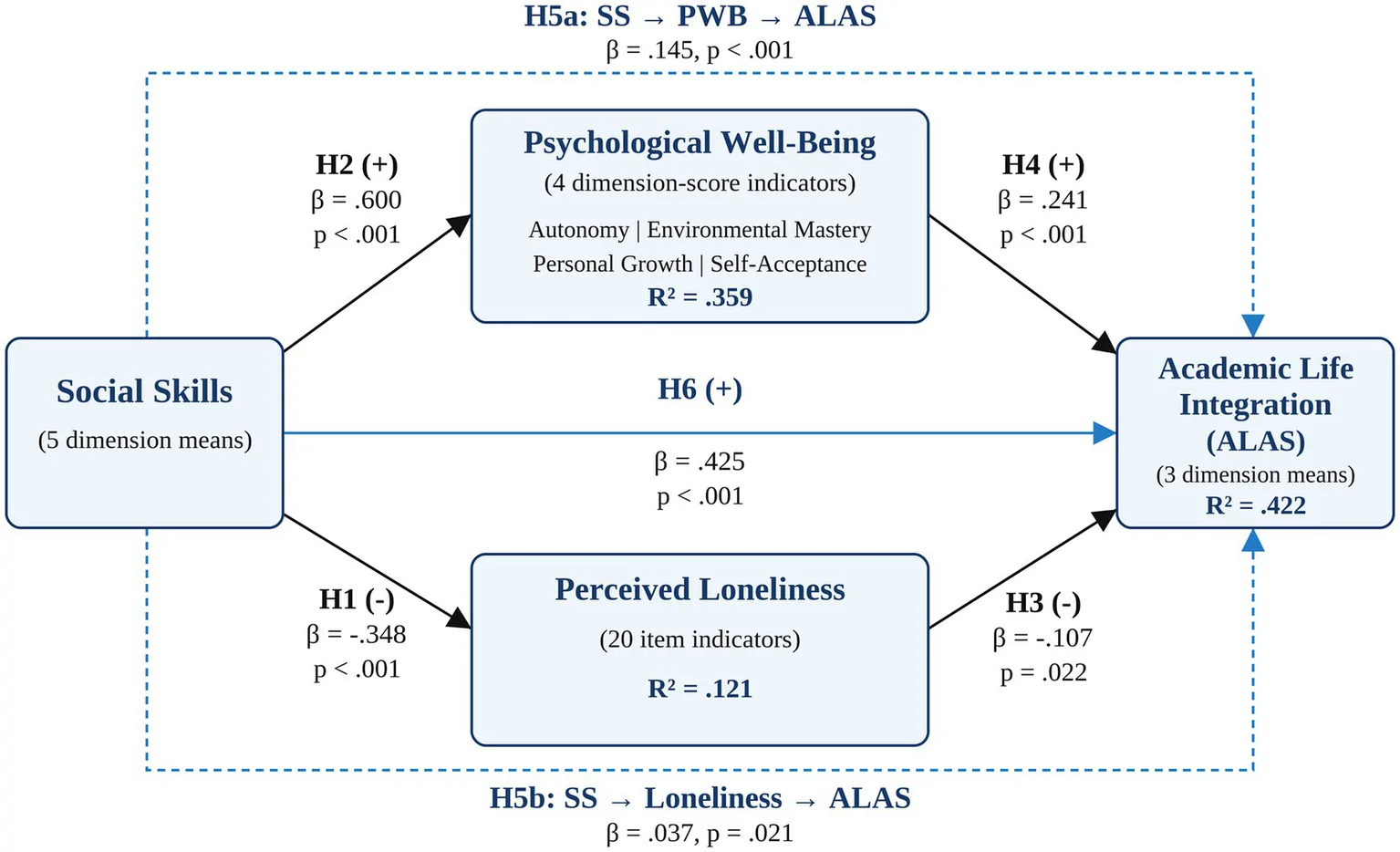
Four-dimensional PWB sensitivity model.

**Table 9:** Structural associations across the primary and sensitivity specifications.

Association	Six dimensions *β* [95% CI]; *p*	Five dimensions *β* [95% CI]; *p*	Four dimensions *β* [95% CI]; *p*
H1: SS → PL	−0.349 [−0.465, −0.238]; *p* < 0.001	−0.349 [−0.465, −0.238]; *p* < 0.001	−0.348 [−0.465, −0.238]; *p* < 0.001
H2: SS → PWB	0.621 [0.533, 0.711]; *p* < 0.001	0.622 [0.534, 0.711]; *p* < 0.001	0.600 [0.501, 0.695]; *p* < 0.001
H3: PL → ALAS	−0.081 [−0.173, 0.015]; *p* = 0.093	−0.083 [−0.173, 0.013]; *p* = 0.087	−0.107 [−0.196, −0.014]; *p* = 0.022
H4: PWB → ALAS	0.284 [0.164, 0.413]; *p* < 0.001	0.280 [0.161, 0.406]; *p* < 0.001	0.241 [0.127, 0.361]; *p* < 0.001
H6: SS → ALAS	0.401 [0.293, 0.499]; *p* < 0.001	0.403 [0.296, 0.502]; *p* < 0.001	0.425 [0.322, 0.522]; *p* < 0.001
H5a: SS → PWB → ALAS	0.176 [0.099, 0.271]; *p* < 0.001	0.174 [0.096, 0.268]; *p* < 0.001	0.145 [0.075, 0.231]; *p* < 0.001
H5b: SS → PL → ALAS	0.028 [−0.006, 0.060]; *p* = 0.090	0.029 [−0.005, 0.061]; *p* = 0.084	0.037 [0.005, 0.069]; *p* = 0.021
Total indirect	0.205 [0.127, 0.298]; *p* < 0.001	0.203 [0.125, 0.296]; *p* < 0.001	0.182 [0.108, 0.271]; *p* < 0.001
Total SS → ALAS	0.606 [0.533, 0.676]; *p* < 0.001	0.606 [0.533, 0.676]; *p* < 0.001	0.607 [0.534, 0.677]; *p* < 0.001

The four-dimensional model explained 42.2% of ALAS variance (*R*^2^ = 0.422; adjusted *R*^2^ = 0.415), compared with 43.0% in the primary model. Its *R*^2^ values were 0.359 for PWB (adjusted 0.357) and 0.121 for loneliness (adjusted 0.118). Four-dimensional *f*^2^ values were 0.017 for PL → ALAS, 0.063 for PWB → ALAS, 0.192 for SS → ALAS, 0.138 for SS → PL, and 0.561 for SS → PWB. The increased statistical support for the loneliness pathway therefore did not coincide with a larger proportion of ALAS variance explained.

#### 4.4.3 Model fit and interpretation

The four-dimensional model had lower SRMR (0.072 saturated; 0.076 estimated), but SRMR, d_ULS and d_G still exceeded their 95th and 99th-percentile bootstrap reference values. Thus, the improved approximate fit did not establish exact fit. Fit statistics were not reported in the five-dimensional bootstrap export.

The sensitivity results preserve the direction of associations but show that their magnitude and statistical support depend on PWB content. Omitting Purpose in Life and Positive Relations narrows the construct; improved reliability or AVE does not establish the reduced model as the correct replacement. The six-dimensional model remains primary. These comparisons do not formally test differences between models or between the two indirect pathways.

## 5 Discussion

The study aimed to explore the relationships among Social Skills, Loneliness, Psychological Well-Being, and academic life integration among students. The primary results are interpreted from the six-dimensional PWB specification, while the five and four-dimensional reductions are used only to evaluate sensitivity to measurement specification.

Social Skills were found to have a direct negative association with loneliness. Hypothesis H1 was supported. Communication, emotional regulation, and conflict resolution provide a plausible account of this association. These skills may support peer relationships, conflict management, and a sense of belonging. Our results are in line with the previous study by Moeller and Seehuus (2019), who linked Social Skills, loneliness, depression, and anxiety in college students. Related research has also examined stress, loneliness, and social support in relation to the association between Social Skills and psychological health (Segrin et al., 2016; Segrin, 2019). These comparisons support the relevance of interpersonal resources without establishing their causal role in the present sample.

Furthermore, our Hypothesis H2, which indicated a positive relationship between Social Skills and Psychological Well-Being, was supported. Our findings are consistent with research linking Social Skills and positive psychological functioning through perceived stress and interpersonal relationships (Segrin et al., 2007; Segrin and Taylor, 2007). From the complementary perspectives of SCT and SDT, interpersonal competence may facilitate access to resources and experiences of relatedness. Positive functioning is nevertheless broader than the absence of loneliness, which supports examining the two pathways separately.

In the primary six-dimensional model, the relationship between loneliness and academic life integration was negative but did not reach statistical significance (*β* = −0.081, *p* = 0.093); Hypothesis H3 was therefore not supported in the primary analysis. Loneliness was negatively correlated with ALAS in the construct-score correlations (*r* = −0.334; Table 3), but its association was smaller and uncertain after accounting for Social Skills and PWB. This attenuation is consistent with overlapping associations among the included predictors; it does not demonstrate that PWB causally explains away loneliness. The result concerns the additional association of loneliness within this parallel model, not an absence of association with university experience.

The direction of this association is consistent with the negative loneliness-engagement relationships reported by Mizani et al. (2022) and Ramli et al. (2024). However, their remote-learning settings and engagement outcomes differ from those of the present study. ALAS includes perceived academic ability and course commitment as well as non-compulsory participation. General feelings of social disconnection may therefore be less closely aligned with the remaining variation in this broader outcome once positive functioning and Social Skills are taken into account. This is a possible explanation rather than a tested difference between learning settings or outcome measures.

Our results support the positive relationship between Psychological Well-Being and academic life integration, corroborating our Hypothesis H4. This is consistent with related research on well-being and engagement (Puiu et al., 2024), while Klapp et al. (2024) examined the related but distinct outcome of academic achievement. Positive functioning may be particularly relevant to an outcome that includes perceived academic ability and course commitment. However, common self-appraisal content and item-wording effects could contribute to this association. Reciprocal well-being-engagement findings (Datu and King, 2018) also caution against assuming a single causal direction.

As expected, the indirect association through Psychological Well-Being was significant. Therefore, Hypothesis H5a was supported in the primary six-dimensional model [*β* = 0.176, 95% CI: (0.099, 0.271), *p* < 0.001], and this central indirect pathway remained significant in the reduced sensitivity specifications. An SCT interpretation is that Social Skills facilitate drawing key resources for improving Psychological Well-Being from social, emotional, and institutional support, and this may be associated with academic life integration. Collaborative learning, exchange of ideas, joint problem-solving, and securing help and feedback may increase an individual’s Self-Efficacy, Sense of Agency, and Autonomy in stressful situations. Favorable relational engagement and peer acceptance may provide students with resources to cope with insecurity and uncertainty and to persist in demanding and intense social and academic contexts. These processes provide a theoretical interpretation, rather than separately tested mechanisms. In an examination of social support, a construct distinct from Social Skills, Yu et al. (2024) found a significant positive effect on Psychological Well-Being. This is in line with the study by Gebregergis et al. (2023), which found that students in higher education with higher levels of psychological capital exhibit better positive psychological functioning and are more engaged in learning. The significant PWB pathway does not reflect equal contributions across all six Ryff dimensions, particularly given the weak Purpose in Life and Positive Relations.

Finally, we posited an indirect association between Social Skills and academic life integration via loneliness. We reasoned that under high demands and intense assignments, students may disengage from group activities and teamwork. Such detachment and lack of social support may decrease their ability to benefit from cooperation and community, leverage resources, seek help, and provide and receive feedback. In the primary six-dimensional model, this pathway was positive but not significant [*β* = 0.028, 95% CI (−0.006, 0.060), *p* = 0.090], so H5b was not supported. The significant social-skills-to-loneliness association alone does not establish an indirect association with ALAS: uncertainty in the conditional loneliness-to-ALAS relationship is reflected in the bootstrap interval for the indirect association. The sensitivity results (Section 4.4) retain the same direction but change statistical support only when Purpose in Life and Positive Relations are both omitted from PWB. Altering the content of PWB may change the allocation of shared variance; this possibility was not directly tested. The findings therefore support neither a definitive null conclusion nor an established loneliness mechanism. Nor does significance of one indirect pathway and non-significance of the other establish a statistically significant difference between them.

The direct association between Social Skills and academic life integration also remained positive after both proposed mediators were included, supporting H6. The two indirect pathways therefore do not account for the full modeled relationship. Social Skills may support greater flexibility, responsiveness, and adaptability across diverse situations and settings. Communication, cooperation, and help-seeking provide a plausible account of this remaining association with participation in university life, rather than evidence of an intervention effect.

## 6 Conclusion and contribution

The current study examined the direct and indirect associations between Social Skills and loneliness, Psychological Well-Being, and academic life integration. In the primary six-dimensional PWB model, Hypotheses H1, H2, H4, H5a, and H6 were supported, whereas Hypotheses H3 and H5b were not. The indirect association through Psychological Well-Being was significant, while the indirect association through loneliness was not significant in the primary model. Five and four-dimensional PWB reductions were examined as sensitivity analyses; the loneliness direct and indirect paths became significant only in the four-dimensional specification. The contribution lies in distinguishing positive psychological functioning from low loneliness within one model of academic life integration. In this sample, the PWB indirect association remained significant across the examined specifications, whereas statistical support for the loneliness pathway depended on the PWB content retained. This supports a context-specific interpretation of the two pathways, not a claim that they are interchangeable or that one is statistically superior.

Besides the development of academic proficiency and knowledge, an educational institution is an environment of social integration and embeddedness, a cross-section of personal, social, and institutional factors (Astin, 1975, 1984, 1997). Accordingly, private and public colleges and universities should foster group participation, involvement, relational connectedness, and trust as possible supports for students’ Resilience, persistence, commitment, and motivation (Browning et al., 2018; Xerri et al., 2018). In practice, educators could pilot structured peer tasks with explicit communication and help-seeking practices, while student support services could combine first-year mentoring with accessible well-being support. Such initiatives should be evaluated using baseline/follow-up assessment and, where feasible, comparison groups. Evaluation should assess perceived academic ability, course commitment and non-compulsory participation alongside well-being and loneliness, rather than assume that improvements in social contact alone produce better academic integration.

The cross-sectional, single-source design limits temporal and causal interpretation and cannot rule out reciprocal associations, confounding or common method bias—non-probability sampling and an uneven demographic profile limit generalization. The primary six-dimensional PWB construct showed weak convergent validity (AVE = 0.444), including an effectively zero Purpose in Life loading and a weak Positive Relations loading. The within-dimensional item screening and subsequent five and four-dimensional sensitivity reductions require independent validation, and the change in H3 and H5b across specifications indicates model-specification uncertainty. Global fit of the primary model was also limited. Longitudinal research, independent measurement validation, and more diverse institutions and learning settings are needed to assess the stability of these associations. The future studies could also examine institutional support, self-efficacy, and social awareness as possible explanations for these relationships; they were not tested as such in the present model.

## Data Availability

The raw data supporting the conclusions of this article will be made available by the authors, without undue reservation.
